# Mother’s Emotional and Posttraumatic Reactions after a Preterm Birth: The Mother-Infant Interaction Is at Stake 12 Months after Birth

**DOI:** 10.1371/journal.pone.0151091

**Published:** 2016-03-29

**Authors:** Anne-Cécile Petit, Julien Eutrope, Aurore Thierry, Nathalie Bednarek, Laurence Aupetit, Stéphanie Saad, Lauriane Vulliez, Daniel Sibertin-Blanc, Sylvie Nezelof, Anne-Catherine Rolland

**Affiliations:** 1 CHU Reims, Hôpital Robert Debré, Service de Psychothérapie de l’Enfant et de l’Adolescent, Reims, France; 2 CHU Reims, Hôpital Robert Debré, Unité d’aide méthodologique, Reims, France; 3 CHU Reims, American-Memorial-Hospital, Service de réanimation néonatale et néonatologie, Reims, France; 4 Centre d’Action Médico-Sociale Précoce, Reims, France; 5 CHU Nancy, Centre psychothérapique de Nancy, Service de pédopsychiatrie, Laxou, France; 6 CHU de Besançon, Hôpital Saint Jacques, Service de psychiatrie de l’enfant et de l’adolescent, Besançon, France; Hôpital Robert Debré, FRANCE

## Abstract

**Objectives:**

Very preterm infants are known to be at risk of developmental disabilities and behavioural disorders. This condition is supposed to alter mother-infant interactions. Here we hypothesize that the parental coping with the very preterm birth may greatly influence mother-infant interactions.

**Methods:**

100 dyads were included in 3 university hospitals in France. Preterm babies at higher risk of neurodevelopmental sequelae (PRI>10) were excluded to target the maternal determinants of mother-infant interaction. We report the follow-up of this cohort during 1 year after very preterm birth, with regular assessment of infant somatic state, mother psychological state and the assessment of mother-infant interaction at 12 months by validated scales (mPPQ, HADS, EPDS, PRI, DDST and PIPE).

**Results:**

We show that the intensity of post-traumatic reaction of the mother 6 months after birth is negatively correlated with the quality of mother-infant interaction at 12 months. Moreover, the anxious and depressive symptoms of the mother 6 and 12 months after birth are also correlated with the quality of mother-infant interaction at 12 months. By contrast, this interaction is not influenced by the initial affective state of the mother in the 2 weeks following birth. In this particular population of infants at low risk of sequelae, we also show that the quality of mother-infant interaction is not correlated with the assessment of the infant in the neonatal period but is correlated with the fine motor skills of the baby 12 months after birth.

**Conclusions:**

This study suggests that mothers’ psychological condition has to be monitored during the first year of very preterm infants’ follow-up. It also suggests that parental interventions have to be proposed when a post-traumatic, anxious or depressive reaction is suspected.

## Introduction

Preterm births (< 37 gestational weeks) and very preterm births (< 32 gestational weeks) have constituted a growing public health issue in developed countries over the past 30 years. Indeed, epidemiologic studies show a growing number of preterm and very preterm births [1] associated with a better survival of preterm infants [2]. This is largely due to medical advances in neonatal intensive care units (NICU) and management of high-risk pregnancy. However, preterm infants are known to be at risk of developing neuromotor disabilities, as well as cognitive and behavioral disorders [3]. In NICU, infant care is a subject of major preoccupation leaving sometimes parental distress in the background. However the impact of a very preterm birth on the parental affective state is of high concern, as it constitutes a stressful event that may lead to post-traumatic stress reactions of the parents [4]. Indeed they have to cope with the fear of the infant’s death, the immaturity of the child and the risk of possible severe handicaps. Moreover, these concerns persist for weeks or months after the birth, constituting a lasting traumatic event with potential retraumatisation experiences.

Moreover, the parents of preterm infants often lack psychological support after the hospital discharge of the baby, although they are at high risk of developing mental disorders such as major depressive disorder, anxiety disorders or post-traumatic stress disorder [5, 6]. Indeed, it has been shown in several studies that mothers of preterm infants report more severe levels of depression and anxiety in the neonatal period than mothers of full-term infants [7, 8]. The premature birth may also affect the emotional state of the mothers more durably, as studies reported persistent depressive symptoms and post-traumatic symptoms one year after birth [9]. More generally, it has been shown that untreated perinatal anxiety has a negative impact on maternal health [10–12].

In most studies, maternal depression and perinatal anxiety have a clearly negative impact on the mother-infant relationship [13, 14]. Studies have examined the effects of maternal depression on the interactive relationship and found links between depression and infant cognitive, emotional and developmental delay [15–18]. Thus, the importance of the parents’ role in the lives of preterm infants and concerns for the developmental outcomes of these high-risk infants is now recognized. Environmental factors have been demonstrated to outweigh medical variables as predictors of cognitive development, language and school performance [19–22] and appear to have a more powerful role in predicting social and cognitive outcomes among premature infants than term babies [23, 24]. This is why promoting the maternal psychological well-being and mother-infant relationship is so crucial. With this goal, some teams develop intervention programs and others create scales to track and measure the quality of the mother-infant interactions [25, 26].

However, while the data are very consistent about the impact of anxio-depressive state of the mothers on mother-infant interaction and infant outcome, only few studies examined the impact of post-traumatic symptoms after a premature birth [27], although this event is known to possibly induce post-traumatic stress symptomatology [5]. Therefore, the aim of our study is to assess the impact of post-traumatic reactions of the mother on the mother-infant interaction during the first year after birth. Our main hypothesis was that emotional and post-traumatic reactions of the mother after a preterm birth may impair mother-infant interaction, even if the baby is in good health. 100 mother-infant dyads with a term inferior to 32 gestational weeks at birth were included at the NICU of 3 university hospitals in France. To follow our hypothesis, premature babies at high risk of neurodevelopmental impairment, ie with high perinatal risk score were excluded of the study. For the 62 dyads followed up to 12 months, our results show that the quality of mother-infant interaction 12 months after birth is correlated with the traumatic reaction of the mother 6 months after birth. Moreover it is correlated with the anxious and depressive symptoms of the mother 6 and 12 months after birth, whatever social support she may benefit from.

## Material and Methods

### Study design

**Fig 1:**
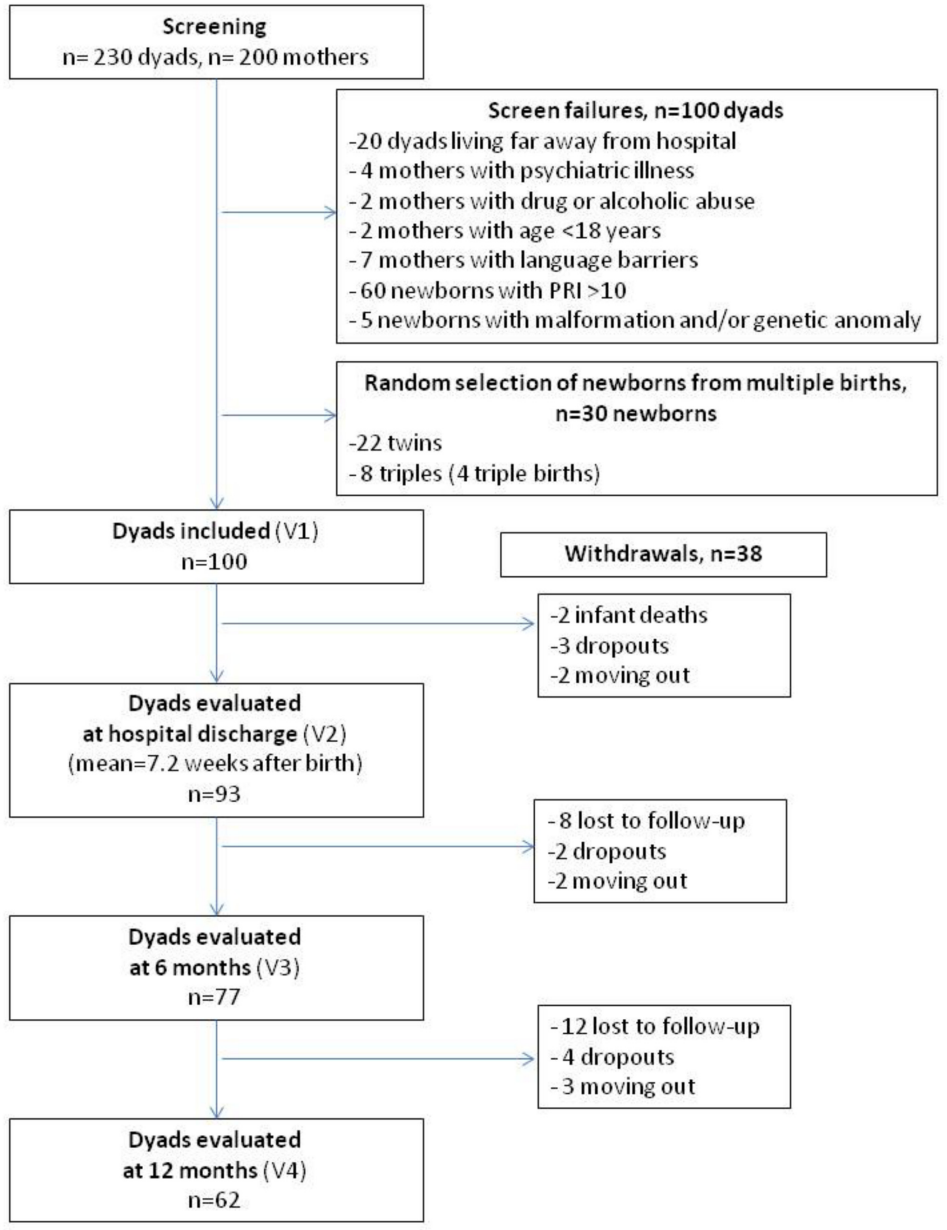
Flowchart of the study. PRI: Perinatal Risk Inventory; V1: assessment at inclusion; V2: assessment at the hospital discharge; V3: assessment 6 months after birth; V4: assessment 12 months after birth.

This study is part of a larger multicenter prospective study performed in 3 French university hospitals (Reims, Nancy and Besançon) between January 2008 and January 2011. The design of the study was already described elsewhere [28]. 100 dyads were included. At their admission to NICU, dyads were screened for inclusion in case of very preterm birth (<32 gestational weeks). Exclusion criteria for the mothers were an evident psychiatric illness, drug abuse, age under 18 and language barrier. For the newborn, unfavorable vital prognosis evaluated with the Perinatal Risk Inventory (PRI, [29]), malformation and/or genetic anomaly diagnosed before the inclusion constituted exclusion criteria. These criteria led to the exclusion of 60 dyads out of 230 screened for inclusion (cf the flowchart presented in Fig 1) and enabled us to select a population of newborns at low risk of neurological sequelae. The entire research program covers 5 visits: the first visit was made in the maternity service within 2 weeks after birth; the second visit, in the NICU, right before the hospital discharge; the three following visits took place at 6, 12 and 18 months, within the framework of the systematic tracking of premature babies in the network of early medicosocial action centers (CAMSP).

This study received the approval of the Institutional Review Board (IRB) of Reims University Hospital for the entire protocol, including multicentric inclusions for a total of 100 dyads. The study was conducted in compliance with good clinical practice, as indicated in the Declaration of Helsinki. Written consent was obtained from all the patients after providing specific information about the study. Patients were free to refuse to take part in the study or to withdraw from it at any stage on simple request, without any alteration to the care provided to them. Data collection was made anonymous.

### Assessment

The assessment of the mother’s psychological condition and of the mother-infant interactions was made by using several validated scales (Table 1) during the different visits. Sociodemographic variables were gathered at inclusion [28].

**Table 1:** Design of the four first visits of the study.

	Place	Date	PPQ	HADS	EPDS	SSQ	PRI	PIPE	DDST
Visit 1	Maternity	Day 1 to day 15		X		X	X		
Visit 2	NICU	Before hospital discharge	X	X	X	X	X		
Visit 3	CAMSP	6 months	X	X	X	X		X	X
Visit 4	CAMSP	12 months	X	X	X	X		X	X

CAMSP: early medicosocial action center; DDST: Denver Developmental Screening Test; EPDS: Edinburgh Postnatal Depression Scale; HADS: Hospital Anxiety and Depression Scale; NICU: neonatal intensive care unit; PIPE: Pediatric infant parent exam; PPQ: Perinatal PTSD Questionnaire; PRI: Perinatal Risk Inventory; SSQ: Social Support Questionnaire.

The evaluation of the mother's traumatic reaction was done by using the modified Perinatal Post-traumatic stress disorder Questionnaire (mPPQ [30, 31]). This self-report questionnaire is specially adapted to the parents of perinatal high-risk children, in order to evaluate the presence of traumatic elements concerning the birth. For this measurement, we decided to use a cut-off score of ≥19 [30], which identifies a high risk of trauma in the maternal population needing specific care (as referral for therapy). We also evaluated the maternal co-morbidity using the Hospital Anxiety and Depression Scale (HADS [32, 33]), which is a self-report questionnaire divided into 2 sub-scales (anxiety and depression HADS sub-scales). It allows episodes of recent anxiety and depression to be evaluated and to attribute an overall score for each one. We have used a cut-off score of 8 or above, which is suggestive of clinically significant anxious (HADS anxiety sub-scale) or depressive (HADS depression sub-scale) symptomatology [34]. The Edinburgh Post-natal Depression Scale (EPDS [35]) consists of a self-report questionnaire that tracks postnatal depression. A score of 12 or above is suggestive of clinically significant depressive symptomatology. The EPDS cutoff has been found to identify major depression in women, with a good sensitivity and specificity [36]. The scales used to evaluate mothers’ emotional state are screening tools that help to detect a risk for traumatic or anxio-depressive states with a high sensitivity [30, 36]. Mothers with abnormal scores were addressed for specific psychological care with psychologists, psychomotor therapists or psychiatrics, who evaluated the need for specific care by clinical evaluation.

The Social Support Questionnaire (SSQ [37]) is a self-report questionnaire, composed of 6 questions. For each question, the mother writes the names of the people of whom she can rely on (maximum 9 people) and sorts them from the most supportive (first position) to the least supportive (last position). The score is obtained by adding the number of people mentioned in each question. The mother gives also a score of satisfaction indicating the level of fulfillment concerning this support.

We identified infants who were at risk of significant developmental abnormalities by using the Perinatal Risk Inventory (PRI, [29]). This scale is composed of 18 items that describe the weight of the perinatal problems and the severity of the perinatal risk, based on perinatal factors such as the Apgar score, the gestational age, the weight or the cranial perimeter. The score can be from 0 to 51. A score >10 identifies infants at risk of significant developmental disabilities. The developmental evaluation of the baby was made using the Denver Developmental Screening Test (DDST, [38]). This test allows estimating the abilities of the child from 0 to 6 years old in various domains of the development (global motricity, language, fine motricity, social contact). PRI and DDST assessment was made by trained pediatricians blind to the results of the questionnaires fulfilled by the mothers.

The mother-infant interaction was evaluated by the Pediatric Infant Parent Exam (PIPE [39]), a screening tool for mother-infant problematic interaction [40, 41]. This scale is usable for infants between 0 and 18 months. The mother is invited to play during a short duration with her baby. The observer of the interaction grants a score to the degree of interactional reciprocity and to positive affect at the beginning, during and at the end of the game. Each of these 3 segments of the game is evaluated on a scale from 1 to 6, the lowest scores being attributed to the more favorable types of interaction. A total score is then calculated by adding the 3 obtained scores. PIPE assessment was made by trained psychologists and psychomotor therapists blind to the results of the questionnaires fulfilled by the mothers.

### Statistical analysis

Normality tests were performed for all variables. Clinical scores with discrete values (PPQ, HADS, EPDS, SSQ, PRI, Apgar and PIPE) did not follow a Gaussian distribution. ANOVA tests were used to compare quantitative normal variables between our sample, the lost of follow-up and the initial population of the study. Kruskal-Wallis rank sum tests were used to compare quantitative non-normal variables in these 3 populations. Chi2 tests were applied to compare qualitative variables according to these 3populations. Paired t-test was applied to compare the corrected age and the developmental age estimated by DDST. Spearman’s rank correlation analysis was used to measure the strength of the relationship between two quantitative variables. A p-value of less than 0.05 was considered statistically significant. Statistical analyses were performed with R environment [42] and Rcmdr interface.

## Results

### Population sample

During the first year of follow-up, 93 dyads were evaluated at the hospital discharge (7.2 ± 3.6 weeks after birth), 77 dyads were evaluated 6 months after birth, and 62 dyads were evaluated 12 months after birth (Fig 1), corresponding to an attrition rate of 38% at 12 months. This attrition was due to infant death (2 infants), lost to follow-up (20 dyads), dropping out of the study (9 dyads) or family moving out (7 dyads). The sample of 62 dyads did not differ from the initial population of 100 dyads and from the 38 dyads who withdrew from the study in term of infants and mothers’ characteristics at the inclusion (Table 2). In the population who withdrew from the study, the mothers tended to have had less C-sections but this is not statistically significant (p = 0.07). At inclusion, 41 infants (41%) were fed with their mother’s breast milk, given by a feeding tube or pipette. 34 (34%) received artificial milk replacer and 25 (25%) received both.

**Table 2:** Socio-demographic and clinical characteristics at inclusion of the 100 dyads included, of the 62 dyads evaluated 12 months after birth and of the 38 dyads lost to follow-up.

Infants’ clinical characteristics	Dyadsincludedn = 100	Dyads evaluatedat 12 monthsn = 62	Lost to follow-upat 12 monthsn = 38	
Gestational age (m ± sd, weeks)	29.6 ± 1.7	29.7 ± 1.6	29.4 ± 1.9	*p = 0*.*74*
Birth weight (m ± sd, grams)	1319 ± 330	1297 ± 344	1357 ± 1.6	*p = 0*.*67*
Sex (nb males, %)	57 (57%)	34 (54.8%)	23 (60.5%)	*p = 0*.*33*
Apgar score at 5 min (Q2, min-max)	8 (0–10)	8 (0–10)	8 (1–10)	*p = 0*.*93*
Apgar score at 10 min (Q2, min-max)	9 (3–10)	9 (3–10)	9 (3–10)	*p = 0*.*98*
Need for neonatal resuscitation* (nb, %)	39 (39%)	25 (40.3%)	14 (36.8%)	*p = 0*.*94*
PRI score (Q2, min-max)	4 (0–10)	4 (0–10)	4 (1–10)	*p = 0*.*87*
Length of hospitalization (m ± sd, weeks)	7.7 ± 2.4	7.8 ± 2.4	7.6 ± 2.6	*p = 0*.*96*
Mothers’ socio-demographic and clinical characteristics				
Age (m ± sd, years)	29.8 ± 5.9	30.4 ± 5.5	28.9 ± 6.5	*p = 0*.*47*
In couple (nb, %)	92 (92%)	57 (92%)	35 (92%)	*p = 0*.*99*
Graduated from high school (nb, %)	74 (74%)	49 (79%)	25 (66%)	*p = 0*.*47*
Higher education (nb, %)	51 (51%)	34 (55%)	15 (39.5%)	*p = 0*.*31*
Nb of pregnancies (m ± sd)	2.1 ± 1.4	1.9 ± 1.3	2.3 ± 1.6	*p = 0*.*27*
Nb of childbirths (m ± sd)	1.8 ± 1.1	1.7 ± 0.9	1.9 ± 1.3	*p = 0*.*63*
In vitro fertilization (nb, %)	10 (10%)	6 (9.6%)	4 (10.5%)	*p = 0*.*99*
Multiple pregnancy (nb, %)	22 (22%)	14 (24%)	8 (21%)	*p = 0*.*87*
Threatened premature labor (nb, %)	76 (76%)	49 (79%)	27 (71%)	*p = 0*.*66*
Caesarean section (nb, %)	54 (54%)	39 (63%)	15 (39.5%)	*p = 0*.*07*
HADS anxiety score (Q2, min-max)	9.5 (1–21)	9.5 (2–21)	9.5 (1–20)	*p = 0*.*99*
HADS depression score (Q2, min-max)	6 (0–19)	7 (0–19)	5.5 (0–19)	*p = 0*.*99*

At the inclusion (visit 1), socio-demographic characteristics were gathered and a first assessment was made.

*Neonatal resuscitation included tube placement and/or cardiac compression and/or epinephrine/vasopressive drug requirement.

m: mean, sd: standard deviation, nb: number, Q2: median or second quartile, min: minimal value, max: maximal value.

At the assessment at 12 months, the mean baby age was 12.67 months ± 1.4, the mean corrected age was 10.22 months ± 1.4. The mean DDST score at 12 months was 11.4 months ± 1.8. 32 infants (52%) were delayed in comparison to their age, with a mean delay of 2.3 months (with a range from 1 to 9 months). 2 infants displayed a developmental delay superior to 6 months. The mean personal-social sub-score was 11.6 ± 2.0, the mean fine motor adaptative sub-score was 11.8 ± 1.7, the mean language sub-score was 11.3 ± 1.9, and the mean gross motor sub-score was 10.8 ± 2.2. The mean DDST score was significantly lower than the mean real age (p = 3.4x10^-6^) and significantly higher than the mean corrected age (p = 2.6x10^-6^).

### Correlation between mother-infant interactions at 12 months and mothers’ clinical characteristics at 6 and 12 months after birth

The median PIPE score at 12 months was 3 (3–6 as 10^th^ and 90^th^ percentiles): 44 dyads (71%) showed a highly adaptative score (3 or 4); 18 dyads (29%) showed a marginally adaptative score (5 to 9), no dyad was assessed with a problematic interaction (score from 10 to 18).

The mothers’ clinical assessment was performed at 6 months and 12 months. At 6 months, the median PPQ score was 15.0 (3.5–28.5 as 10^th^ and 90^th^ percentiles). 31 mothers (40.2%) showed a score equal or above 19 (from 19 to 46), which corresponds to a risk of post-traumatic stress reaction. The median HADS anxiety score was 6.5 (3.5–11 as 10^th^ and 90^th^ percentiles). 32 mothers (41.5%) showed an anxiety score equal to or above 8 (from 8 to 14), which corresponds to a significant anxious symptomatology. The median HADS depression score was 3.0 (0–8.5 as 10^th^ and 90^th^ percentiles). 9 mothers (11.7%) showed a depression score equal to or above 8 (from 8 to 11), which corresponds to a significant depressive symptomatology. The median EPDS score was 6.0 (1–11 as 10^th^ and 90^th^ percentiles). 7 mothers (9.1%) showed a score equal to or above 12 (from 12 to 16), corresponding to a significant post-natal depressive symptomatology.

At 12 months, the median PPQ score was 12.0 (2–26 as 10^th^ and 90^th^ percentiles). 21 mothers (34%) showed a score equal to or above 19 (from 19 to 67). The median HADS anxiety score was 6.0 (3–10 as 10^th^ and 90^th^ percentiles). 24 mothers (39%) showed an anxiety score equal or above 8 (from 8 to 21). The median HADS depression score was 3.0 (0–11 as 10^th^ and 90^th^ percentiles). 13 mothers (21%) showed a depression score equal to or above 8 (from 8 to 16). The median EPDS score at 12 months was 5.0 (1–16 as 10^th^ and 90^th^ percentiles). 12 mothers (19%) showed a score equal to or above 12 (from 12 to 25).

A positive correlation is found between PIPE score at 12 months and PPQ score assessing the mother’s post-traumatic stress reaction 6 months after birth (Table 3A). A trend is also observed towards a correlation between PIPE score at 12 months and PPQ score at 12 months and at the hospital discharge (Table 3A).

**Table 3:** Correlations between PIPE score at 12 months and mother’s and baby’s clinical characteristics.

A. Mother’s clinical characteristics	Correlation with PIPE score 12 months after birth	B. Baby’s clinical characteristics	Correlation with PIPE score 12 months after birth
Age	NS	Intrauterine growth restriction	NS
Multiple pregnancy	NS	Term of birth	NS
Threatened premature labor	NS	Weight at birth	NS
		Apgar score	
C-section	NS	5 min	NS
History of baby loss	NS	10 min	NS
PPQ			
V2	0.22 (p = 0.10)	Hospitalization duration	NS
		PRI	
V3	0.34 (p = 0.008*)	V1	NS
V4	0.21 (p = 0.11)	V2	NS
HADS anxiety score			
		DDST score	
V1	NS	V4	-0.19 (p = 0.15)
		DDST personal-social subscore	
V2	NS	V4	-0.18 (p = 0.14)
		DDST language subscore	
V3	0.25 (p = 0.05)	V4	-0.18 (p = 0.16)
		DDST fine motor adaptative subscore	
V4	0.29 (p = 0.02*)	V4	-0.28 (p = 0.03*)
HADS depression score		DDST gross motor subscore	
V1	NS	V4	-0.19 (p = 0.14)
V2	NS		
V3	0.22 (p = 0.09)		
V4	0.30 (p = 0.02*)		
EPDS			
V2	NS		
V3	0.31 (p = 0.01*)		
V4	0.43 (p = 0.0006*)		
SSQ number score			
V1	NS		
V2	NS		
V3	NS		
V4	NS		

Correlation is estimated with ρ Spearman coefficient. DDST: Denver Developmental Screening Test; EPDS: Edinburgh Postnatal Depression Scale; HADS: Hospital Anxiety and Depression Scale; PIPE: Pediatric infant parent exam; PPQ: Perinatal PTSD Questionnaire; PRI: Perinatal Risk Inventory; SSQ: Social Support Questionnaire; min: minute; NS: not significant; V1: assessment at inclusion; V2: assessment at the hospital discharge; V3: assessment 6 months after birth; V4: assessment 12 months after birth.

*: p<0.05.

The PIPE score at 12 months was correlated with HADS anxiety scale score and EPDS score at 6 months and HADS anxiety scale score, HADS depression scale score and EPDS score at 12 months (Table 3A). It was not correlated with the initial assessment of anxiety in mothers by HADS anxiety scale 2 weeks after birth or at the hospital discharge or with the initial assessment of mother depressive state by HADS depression scale 2 weeks after birth or by HADS depression scale and EPDS at the hospital discharge.

Regarding the social support, the partner, ie the father of the baby, was mentioned by the mothers as a supportive person in 71.4% of the cases. The partner was mentioned in first position in 65% of the cases. The other people mentioned by the mothers in first position were their parents, ie the maternal grand-parents of the baby in 18.8% of the cases (as follows: the 2 parents in 11.2% of the cases, only the mother in 6.6% and only the father in 1%) and other people in 16.2% of the cases (including their brother or sister, their friends and their parents in-law ie the paternal grand-parents of the baby). No correlation was observed between the PIPE score at 12 months and SSQ scores (SSQ number score and SSQ satisfaction score) at any assessment time.

The PIPE score at 12 months was not correlated with the obstetrical history of the mother (Table 3A).

### Correlation between mother-infant interactions at 12 months and babies’ clinical characteristics

A trend was observed towards a negative correlation between PIPE score at 12 months and DDST score at 12 months (Table 3B). This trend was also observed between PIPE score and DDST sub-scores, with a significant correlation between PIPE score at 12 months and the fine motor adaptative sub-score (ρ = -0.28, p = 0.03*, Table 3B).

The PIPE score at 12 months was not correlated with the initial clinical characteristics of the baby (Table 3B). No correlation was observed between the PIPE score at 12 months and the perinatal risk assessed by the PRI score 2 weeks after birth and at the hospital discharge (Table 3B). To note, DDST score at 12 months was not correlated with PRI score 2 weeks after birth (p = 0.94); a trend towards a correlation between DDST score at 12 months and PRI at the hospital discharge was observed (ρ = 0.22, p = 0.15).

### Factors influencing mothers’ traumatic reaction 6 months after birth

Given that the PIPE score is influenced by the mother’s post-traumatic stress reaction 6 months after birth, we aimed to determine the factors correlated with this traumatic reaction.

The PPQ score at 6 months was positively correlated with the delivery conditions (Table 4A). It was also correlated with the anxio-depressive state of the mother at the inclusion, at the hospital discharge and 6 months after birth (Table 4A). As for the baby’s clinical characteristics, the PPQ score at 6 months was correlated with the intrauterine growth restriction and with the PRI score at inclusion (Table 4B).

**Table 4:** Correlations between PPQ score at 6 months and mother’s and baby’s clinical characteristics.

A. Mother’s clinical characteristics	Correlation with PPQ 6 months after birth	B. Baby’s clinical characteristics	Correlation with PPQ 6 months after birth
Age	NS	Intrauterine growth restriction	0.25 (p = 0.03*)
Multiple pregnancy	NS	Term of birth	NS
History of baby loss	NS	Weight at birth	NS
		Apgar score	
Threatened premature labor	NS	5 min	NS
C section	0.28 (p = 0.01*)	10 min	NS
PPQ			
V2	0.63 (p = 2.51x10^-9^*)	Hospitalization duration	NS
HADS anxiety score			
		PRI	
V1	0.44 (p = 7.82x10^-5^*)	V1	0.23 (p = 0.04*)
V2	0.25 (p = 0.03*)	V2	0.23 (p = 0.08)
V3	0.45 (p = 3.92x10^-5^*)		
HADS depression score			
V1	0.29 (p = 0.01*)		
V2	0.24 (p = 0.04*)		
V3	0.33 (p = 0.003*)		
EPDS			
V2	0.34 (p = 0.003*)		
V3	0.50 (p = 3.81x10^-6^*)		
SSQ number score			
V1	NS		
V2	NS		
V3	NS		

Correlation is estimated with ρ Spearman coefficient. EPDS: Edinburgh Postnatal Depression Scale; HADS: Hospital Anxiety and Depression Scale; PPQ: Perinatal PTSD Questionnaire; PRI: Perinatal Risk Inventory; SSQ: Social Support Questionnaire; min: minute; NS: not significant; V1: assessment at inclusion; V2: assessment at the hospital discharge; V3: assessment 6 months after birth.

*: p<0.05

To note, the PPQ score at 12 months was also found to be correlated with delivery conditions and was highly correlated with the scores of depression and anxiety HADS and EPDS at all assessment times (S1 Table). By contrast, no significant correlation was found with the PPQ score at 12 months and the baby’s clinical characteristics.

## Discussion

Our study aimed at evaluating the impact of mothers’ traumatic reaction on the mother-infant interactions 12 months after a preterm birth, in a population of preterm babies at low risk of neurodevelopmental sequelae, to target the maternal determinants of mother-infant interaction. We showed that the traumatic reaction at 6 months and clinically significant anxious and depressive symptoms experienced by the mother at 6 and 12 months after birth are correlated to the quality of mother-infant interactions at 12 months. However we showed that anxious or depressive symptoms of the mother during the neonatal period are not correlated to the quality of mother-infant interactions at 12 months. We also found that, in a population of premature infants with low perinatal risk, the initial characteristics and the initial evaluation of the perinatal risk of the infant are not correlated with the quality of mother-infant interactions at 12 months.

Our study aimed at exploring factors other than the possible neurodevelopmental sequelae of the preterm infants [43] that may play a predominant role in mother-infant interaction over the first years of the baby’s life. In a population of infants with low risk of sequelae, it appears that the quality of mother-infant interaction at 12 months is not correlated with the initial clinical characteristics of the baby and with the PRI scores. It is noteworthy that the babies in our sample displayed a mean average developmental delay of 2.3 months at 12 months, with a range from 1 to 9 months. This shows that the majority of the babies in our sample displayed a satisfying development. However, even if PRI at birth was low, 2 babies in this sample have important developmental delay (superior to 6 months). Of note, the 2 dyads concerned did not display problematic mother-infant interaction. This may be due to the limited number of dyads with important developmental delay. Moreover, the DDST is known to be less efficient to detect minor developmental problems that may impact the mother-infant interactions. However, we found that the quality of mother-infant interaction at 12 months is correlated with the fine motor adaptative subscore of the DDST (Table 3B). This result may indicate that the fine motor skills of the baby, like grasping or clapping, are particularly significant for the mother-infant interaction. This is in line with other studies that demonstrate that the mother's behavior is influenced by the 12-months-infant's object manipulation [44].

Our results show that the quality of mother-infant interaction at 12 months is correlated with the post-traumatic reaction of the mother 6 months after birth (Table 3A). A trend towards a correlation between mother-infant interaction at 12 months and the post-traumatic reaction of the mother 12 months after birth is also observed. Very few studies have examined the influence of traumatic reaction of the mother on the quality of mother-infant interaction but some results suggest cognitive biases towards specific infant emotions, thus influencing this interaction [45].

It appears that the traumatic reaction of the mothers is highly correlated with the anxio-depressive state of the mother at all assessment times (Table 4A and S1 Table) but not with the characteristics of the baby (Table 4B and S1 Table). This result is in line with several studies, showing that possible parents’ post-traumatic stress disorder was not associated with the severity of the infant’s illness [46] and that the traumatic reaction was not related to infant characteristics (like gestational age, birth weight, Apgar scores, or length of stay in the NICU), but rather to prolonged uncertainty, disruptions in meaning systems and alterations in parental role expectations [47]. The traumatic reaction is also linked to the obstetrical history of the mother: indeed, a correlation is observed between the PPQ score at 6 months and 12 months and C-section delivery and between the PPQ score at 6 months and the presence of an intra-uterine growth restriction. The traumatic reaction at 6 months is also correlated with the PRI score assessed during the first week after the baby’s birth. We may hypothesize that the announcement of a high perinatal risk is a source of heavy stress for the parents, which may be deleterious for parental affective state, although the evolution of the possible sequelae is uncertain.

It also clearly appears in our results that the mother-infant interaction at 12 months is correlated with the anxious and depressive state of the mother 6 months after birth and 12 months after birth. This is in line with some studies suggesting that mothers with depression and anxiety are more likely to identify negative emotions in their infant’s facial expressions and that they may disengage faster from positive and negative infant emotional expressions [45]. Of note, the mother-infant interaction at 12 months is not affected by the initial anxio-depressive state of the mothers (at birth and at the hospital discharge). This result may suggest that the affective state of the mothers particularly impact mother-infant interaction after the return back home, possibly associated with less medical and paramedical support.

It is noteworthy that the quality of mother-infant interaction and the traumatic reaction of the mother are independent of the social support experienced by the mothers in our study. This stands in contrast with other studies showing that social support might buffer against the potentially traumatic effect of an emergency C-section [48].

This study has several strengths: the 12-months follow-up enables us to monitor mother-infant interaction over a large period of time; the attrition rate is quite low; the sample size is relatively high comparing to other studies (that classically includes 30 to 70 dyads with preterm birth [27, 46, 49]); we used a panel of validated scales to evaluate both infants and mothers over time. This study also has some limitations: firstly, the assessment of mother-infant interaction at 12 months shows no dyad with problematic interaction, which may be explained by the exclusion of premature babies at high risk of neurodevelopmental impairment from the study but may also be due to the sample size; secondly, we lack a control group of dyads with full-term birth, which limits the value of our results; thirdly, the statistical analysis was limited by the non-Gaussian distribution of the clinical scores and the use of non-parametric tests in bivariate analyses, which prevents us to build regression models to better determine the influence of the different factors in the mother-child interactions.

To conclude, nowadays parental interventions are proposed to help mothers facing premature birth and develop adaptative parental caring for the preterm infant [50], in particular when the preterm infant develops major complications related to preterm birth. Our results suggest that these parental interventions have to be proposed according to the mothers’ psychological assessment and the mothers’ obstetrical history. Indeed, it appears that the monitoring of the traumatic reaction and the anxio-depressive state of the mother at 6 and 12 months may represent a very helpful element to predict the quality of mother-infant interaction. This may be done with relatively simple scales (PPQ, HADS and EPDS). Moreover, if the obstetrical history includes a C-section delivery or an intra-uterine growth restriction, or if an announcement of potential neurodevelopmental sequelae is made to the mother just after the birth (whatever the evolution of the baby’s abilities in the following months is), it may also be important to propose a psychological support for the parents.

## Supporting Information

S1 Table Correlations between PPQ score at 6 months and mother’s and baby’s clinical characteristics. (DOCX)

## References

[1] Brady E, Hamilton D, Joyce A. Birth: preliminary data for 2005. Hyattsville: National Center for Health Statistics (NCHS) 2006. 17432301

[2] Fanaroff AA, Stoll BJ, Wright LL, Carlo WA, Ehrenkranz RA, Stark AR, et al Trends in neonatal morbidity and mortality for very low birthweight infants. American journal of obstetrics and gynecology. 2007;196(2):147 e1–8. Epub 2007/02/20. 10.1016/j.ajog.2006.09.014 . 17306659

[3] de Kleine MJ, den Ouden AL, Kollee LA, Ilsen A, van Wassenaer AG, Brand R, et al Lower mortality but higher neonatal morbidity over a decade in very preterm infants. Paediatric and perinatal epidemiology. 2007;21(1):15–25. Epub 2007/01/24. 10.1111/j.1365-3016.2007.00780.x . 17239175

[4] Pierrehumbert B, Nicole C, Muller-Nix C, Forcada-Guex M, Ansermet F. Parental post-traumatic reactions after premature birth: implications for sleeping and eating problems in the infant. Arch Dis Child Fetal Neonatal Ed. 2003;88(5):F400–4. 12937044 10.1136/fn.88.5.F400 PMC1721611

[5] Kersting A, Dorsch M, Wesselmann U, Ludorff K, Witthaut J, Ohrmann P, et al Maternal posttraumatic stress response after the birth of a very low-birth-weight infant. Journal of psychosomatic research. 2004;57(5):473–6. Epub 2004/12/08. 10.1016/j.jpsychores.2004.03.011 . 15581651

[6] Misund AR, Nerdrum P, Braten S, Pripp AH, Diseth TH. Long-term risk of mental health problems in women experiencing preterm birth: a longitudinal study of 29 mothers. Annals of general psychiatry. 2013;12(1):33 Epub 2013/11/02. 10.1186/1744-859x-12-33 24176131 PMC4175092

[7] Davis L, Edwards H, Moyah H, Wollin J. The impact of very premature birth on the psychological health of mothers. Early human development. 2003;73(1–2):61–70. 12932894 10.1016/s0378-3782(03)00073-2

[8] Stjernqvist KM. Extremely low birth weight infants less than 901 g. Impact on the family during the first year. Scandinavian journal of social medicine. 1992;20(4):226–33. Epub 1992/12/01. . 1475650 10.1177/140349489202000407

[9] Garel M, Dardennes M, Blondel B. Mothers' psychological distress 1 year after very preterm childbirth. Results of the EPIPAGE qualitative study. Child: care, health and development. 2007;33(2):137–43. Epub 2007/02/13. 10.1111/j.1365-2214.2006.00663.x . 17291317

[10] Johnson RC, Slade P. Obstetric complications and anxiety during pregnancy: is there a relationship? Journal of psychosomatic obstetrics and gynaecology. 2003;24(1):1–14. Epub 2003/04/11. . 12685335 10.3109/01674820309042796

[11] Matthey S, Barnett B, Howie P, Kavanagh DJ. Diagnosing postpartum depression in mothers and fathers: whatever happened to anxiety? Journal of affective disorders. 2003;74(2):139–47. Epub 2003/04/23. . 12706515 10.1016/s0165-0327(02)00012-5

[12] Milgrom J, Gemmill AW, Bilszta JL, Hayes B, Barnett B, Brooks J, et al Antenatal risk factors for postnatal depression: a large prospective study. Journal of affective disorders. 2008;108(1–2):147–57. Epub 2007/12/11. 10.1016/j.jad.2007.10.014 . 18067974

[13] Neri E, Agostini F, Salvatori P, Biasini A, Monti F. Mother-preterm infant interactions at 3 months of corrected age: influence of maternal depression, anxiety and neonatal birth weight. Frontiers in psychology. 2015;6:1234 Epub 2015/09/22. 10.3389/fpsyg.2015.01234 ; PubMed Central PMCID: PMCPmc4554962. 26388792 PMC4554962

[14] Stevenson-Hinde J, Shouldice A, Chicot R. Maternal anxiety, behavioral inhibition, and attachment. Attachment & human development. 2011;13(3):199–215. Epub 2011/04/21. 10.1080/14616734.2011.562409 . 21506027

[15] Bergman K, Sarkar P, Glover V, O'Connor TG. Maternal prenatal cortisol and infant cognitive development: moderation by infant-mother attachment. Biological psychiatry. 2010;67(11):1026–32. Epub 2010/03/02. 10.1016/j.biopsych.2010.01.002 ; PubMed Central PMCID: PMCPmc2872196. 20188350 PMC2872196

[16] Huhtala M, Korja R, Lehtonen L, Haataja L, Lapinleimu H, Rautava P. Associations between parental psychological well-being and socio-emotional development in 5-year-old preterm children. Early human development. 2014;90(3):119–24. Epub 2014/01/15. 10.1016/j.earlhumdev.2013.12.009 . 24418104

[17] Mayberry LJ, Affonso DD. Infant temperament and postpartum depression: a review. Health care for women international. 1993;14(2):201–11. Epub 1993/03/01. 10.1080/07399339309516041 . 8509323

[18] O'Connor MJ, Shah B, Whaley S, Cronin P, Gunderson B, Graham J. Psychiatric illness in a clinical sample of children with prenatal alcohol exposure. The American journal of drug and alcohol abuse. 2002;28(4):743–54. Epub 2002/12/21. . 12492268 10.1081/ada-120015880

[19] Beaino G, Khoshnood B, Kaminski M, Marret S, Pierrat V, Vieux R, et al Predictors of the risk of cognitive deficiency in very preterm infants: the EPIPAGE prospective cohort. Acta paediatrica (Oslo, Norway: 1992). 2011;100(3):370–8. Epub 2011/01/19. 10.1111/j.1651-2227.2010.02064.x ; PubMed Central PMCID: PMCPmc3080666. 21241364 PMC3080666

[20] Brown HK, Speechley KN, Macnab J, Natale R, Campbell MK. Mild prematurity, proximal social processes, and development. Pediatrics. 2014;134(3):e814–24. Epub 2014/08/13. 10.1542/peds.2013-4092 . 25113289

[21] Schneider LA, Burns NR, Giles LC, Higgins RD, Nettelbeck TJ, Ridding MC, et al Cognitive abilities in preterm and term-born adolescents. The Journal of pediatrics. 2014;165(1):170–7. Epub 2014/05/06. 10.1016/j.jpeds.2014.03.030 . 24793204

[22] Velikos K, Soubasi V, Michalettou I, Sarafidis K, Nakas C, Papadopoulou V, et al Bayley-III scales at 12 months of corrected age in preterm infants: Patterns of developmental performance and correlations to environmental and biological influences. Research in developmental disabilities. 2015;45–46:110–9. Epub 2015/08/02. 10.1016/j.ridd.2015.07.014 . 26232203

[23] Greenberg MT, Crnic KA. Longitudinal predictors of developmental status and social interaction in premature and full-term infants at age two. Child development. 1988;59(3):554–70. Epub 1988/06/01. . 3383667 10.1111/j.1467-8624.1988.tb03216.x

[24] Schraeder BD, Heverly MA, O'Brien C. The influence of early biological risk and the home environment on nine-year outcome of very low birth weight. The Canadian journal of nursing research = Revue canadienne de recherche en sciences infirmieres. 1996;28(4):79–95. Epub 1996/01/01. . 9128477

[25] Milgrom J, Newnham C, Anderson PJ, Doyle LW, Gemmill AW, Lee K, et al Early sensitivity training for parents of preterm infants: impact on the developing brain. Pediatric research. 2010;67(3):330–5. Epub 2009/12/03. 10.1203/PDR.0b013e3181cb8e2f . 19952869

[26] White-Traut R, Norr KF, Fabiyi C, Rankin KM, Li Z, Liu L. Mother-infant interaction improves with a developmental intervention for mother-preterm infant dyads. Infant behavior & development. 2013;36(4):694–706. Epub 2013/08/22. 10.1016/j.infbeh.2013.07.004 ; PubMed Central PMCID: PMCPmc3858517. 23962543 PMC3858517

[27] Forcada-Guex M, Borghini A, Pierrehumbert B, Ansermet F, Muller-Nix C. Prematurity, maternal posttraumatic stress and consequences on the mother-infant relationship. Early human development. 2011;87(1):21–6. Epub 2010/10/19. 10.1016/j.earlhumdev.2010.09.006 . 20951514

[28] Eutrope J, Thierry A, Lempp F, Aupetit L, Saad S, Dodane C, et al Emotional reactions of mothers facing premature births: study of 100 mother-infant dyads 32 gestational weeks. PloS one. 2014;9(8):e104093 Epub 2014/08/26. 10.1371/journal.pone.0104093 25153825 PMC4143228

[29] Scheiner A, Sexton M. Prediction of developmental outcome using a perinatal risk inventory. Pediatrics. 1991;88(6):1135–43. 1720234

[30] Callahan J, Borja S, Hynan M. Modification of the Perinatal PTSD Questionnaire to enhance clinical utility. J Perinatol. 2006;26(9):533–9. 16826190 10.1038/sj.jp.7211562

[31] Pierrehumbert B, Borghini A, Forcada-Guex M, Jaunin L, Muller-Nix C, Ansermet F. French validation of the "Perinatal PTSD Questionnaire" assessing parent's posttraumatic stress reactions following the birth of a high risk infant. Ann Médico-psychol. 2004;162(9):711–21.

[32] Lepine JP, Godchau M, Brun P, Lemperière T. Evaluation de l'anxiété et de la dépression chez des patients hospitalisés dans un service de médecine interne. Ann Médico-Psychol. 1985 2:175–89. 4037594

[33] Zigmond A, Snaith R. The hospital anxiety and depression scale. Acta psychiatr scand. 1983;67(6):361–70. 6880820 10.1111/j.1600-0447.1983.tb09716.x

[34] Hinz A, Brahler E. Normative values for the hospital anxiety and depression scale (HADS) in the general German population. Journal of psychosomatic research. 2011;71(2):74–8. Epub 2011/07/20. 10.1016/j.jpsychores.2011.01.005 . 21767686

[35] Cox JL, Holden JM, Sagovsky R. Detection of postnatal depression: development of the 10-item Edimburgh Postnatal Depression Scale. Br J Psychiatry. 1987;150:782–6. 3651732 10.1192/bjp.150.6.782

[36] Holden JM. Postnatal depression: its nature, effects, and identification using the Edinburgh Postnatal Depression scale. Birth (Berkeley, Calif). 1991;18(4):211–21. Epub 1991/12/01. . 1764150 10.1111/j.1523-536x.1991.tb00104.x

[37] Sarason IG, Levine HM, Basham RB, Sarason BR. Assessing social support: the social support questionnaire. J Personnal Soc Psychol. 1983;44(1):127–39.

[38] Frankenburg W, Dodds J. The Denver develpmental screening test. The Journal of pediatrics. 1967;71(2):181–91. 6029467 10.1016/s0022-3476(67)80070-2

[39] Fiese B, Poehlman J, Irwin M, Curry-Bleggi E. A pediatric screening instrument to detect problematic infant-parent interactions: initial realiability and validity in a sample of high and low risk infant. IMHJ. 2001;22(4):463–78.

[40] Reddy PD, Desai G, Hamza A, Karthik S, Ananthanpillai ST, Chandra PS. Enhancing Mother Infant Interactions through Video Feedback Enabled Interventions in Women with Schizophrenia: A Single Subject Research Design Study. Indian journal of psychological medicine. 2014;36(4):373–7. Epub 2014/10/23. 10.4103/0253-7176.140702 ; PubMed Central PMCID: PMCPmc4201788. 25336768 PMC4201788

[41] Sridaromont KL. Interrater reliability of the Pediatric Infant Parent Exam: Nursing screening as a component of well-baby visits: Texas Woman's University; 2012.

[42] R Core Team T. R: A language and environment for statistical computing.: R Foundation for Statistical Computing, Vienna, Austria; 2013 Available from: http://www.R-project.org/.

[43] Cacciani L, Di Lallo D, Piga S, Corchia C, Carnielli V, Chiandotto V, et al Interaction of child disability and stressful life events in predicting maternal psychological health. Results of an area-based study of very preterm infants at two years corrected age. Research in developmental disabilities. 2013;34(10):3433–41. Epub 2013/08/08. 10.1016/j.ridd.2013.07.018 . 23920026

[44] Fukuyama H, Qin S, Kanakogi Y, Nagai Y, Asada M, Myowa-Yamakoshi M. Infant's action skill dynamically modulates parental action demonstration in the dyadic interaction. Developmental science. 2014 Epub 2014/12/09. 10.1111/desc.12270 . 25483121

[45] Webb R, Ayers S. Cognitive biases in processing infant emotion by women with depression, anxiety and post-traumatic stress disorder in pregnancy or after birth: A systematic review. Cognition & emotion. 2015;29(7):1278–94. Epub 2014/12/05. 10.1080/02699931.2014.977849 . 25472032

[46] Mehler K, Mainusch A, Hucklenbruch-Rother E, Hahn M, Hunseler C, Kribs A. Increased rate of parental postpartum depression and traumatization in moderate and late preterm infants is independent of the infant's motor repertoire. Early human development. 2014;90(12):797–801. Epub 2014/12/03. 10.1016/j.earlhumdev.2014.09.008 . 25463823

[47] Lasiuk GC, Comeau T, Newburn-Cook C. Unexpected: an interpretive description of parental traumas' associated with preterm birth. BMC pregnancy and childbirth. 2013;13 Suppl 1:S13 Epub 2013/03/06. 10.1186/1471-2393-13-s1-s13 23445715 PMC3561145

[48] Noyman-Veksler G, Herishanu-Gilutz S, Kofman O, Holchberg G, Shahar G. Post-natal psychopathology and bonding with the infant among first-time mothers undergoing a caesarian section and vaginal delivery: sense of coherence and social support as moderators. Psychology & health. 2015;30(4):441–55. Epub 2014/10/18. 10.1080/08870446.2014.977281 . 25325733

[49] Agostini F, Neri E, Dellabartola S, Biasini A, Monti F. Early interactive behaviours in preterm infants and their mothers: influences of maternal depressive symptomatology and neonatal birth weight. Infant behavior & development. 2014;37(1):86–93. Epub 2014/01/28. 10.1016/j.infbeh.2013.12.003 . 24463339

[50] Steinhardt A, Hinner P, Kuhn T, Roehr CC, Rudiger M, Reichert J. Influences of a dedicated parental training program on parent-child interaction in preterm infants. Early human development. 2015;91(3):205–10. Epub 2015/02/14. 10.1016/j.earlhumdev.2015.01.012 . 25676187

