# Supplementary material for: Mother’s Emotional and Posttraumatic Reactions after a Preterm Birth: The Mother-Infant Interaction Is at Stake 12 Months after Birth
Source: PLoS One. 2016 Mar 29;11(3):e0151091. doi: 10.1371/journal.pone.0151091 (PMC4811536; doi:10.1371/journal.pone.0151091)
Supplement: S1 Table — (DOCX) [file pone.0151091.s001.docx]

**S1 Table. Correlations between PPQ score at 6 months and mother’s and baby’s clinical characteristics.**

| ***A. Mother’s clinical characteristics*** | | Correlation with PPQ 12 months after birth | ***B. Baby’s clinical characteristics*** |  | Correlation with PPQ 12 months after birth |
| --- | --- | --- | --- | --- | --- |
| Age |  | NS | Intrauterine growth restriction |  | 0.21 (p=0.09) |
| Multiple pregnancy |  | NS | Term of birth |  | NS |
| History of baby loss |  | NS | Weight at birth |  | NS |
| Threatened premature labor |  | NS | Apgar score | 5 min | NS |
| Delivery conditions |  | 0.33 (p=0.009*) |  | 10 min | NS |
| PPQ | V2 | 0.54 (p=1.59x10^-5^*) | Hospitalization duration |  | 0.22 (p=0.08) |
|  | V3 | 0.74 (p=7.4x10^-12^*) | PRI | V1 | 0.24 (p=0.06) |
| HADS anxiety score | V1 | 0.49 (p=4.3x10^-5^*) |  | V2 | NS |
|  | V2 | 0.36 (p=0.005*) | DDST score | V4 | NS |
|  | V3 | 0.46 (p=0.0001*) | DDST personal-social subscore | V4 | NS |
|  | V4 | 0.63 (p=4.5x10^-8^*) | DDST language subscore | V4 | NS |
| HADS depression score | V1 | 0.49 (p=4.34x10^-5^*) | DDST fine motor adaptative subscore | V4 | NS |
|  | V2 | 0.35 (p=0.007*) | DDST gross motor subscore | V4 | NS |
|  | V3 | 0.45 (p=0.0002*) |  |  |  |
|  | V4 | 0.55 (p=3.39x10^-6^*) |  |  |  |
| EPDS | V2 | 0.35 (p=0.007*) |  |  |  |
|  | V3 | 0.50 (p=2.96x10^-5^*) |  |  |  |
|  | V4 | 0.65 (p=1.3x10^-8^*) |  |  |  |
| SSQ number score | V1 | NS |  |  |  |
|  | V2 | NS |  |  |  |
|  | V3 | NS |  |  |  |
|  | V4 | NS |  |  |  |

Correlation is estimated with ρ Spearman coefficient. DDST: [Denver Developmental Screening Test](http://en.wikipedia.org/wiki/Denver_Developmental_Screening_Test); EPDS: [Edinburgh Postnatal Depression Scale](http://www.fresno.ucsf.edu/pediatrics/downloads/edinburghscale.pdf); HADS: Hospital Anxiety and Depression Scale; PPQ: Perinatal PTSD Questionnaire; PRI: Perinatal Risk Inventory; SSQ: Social Support Questionnaire; min : minute ; NS: not significant ; V1 : assessment at inclusion ; V2 : assessment at the hospital discharge ; V3 : assessment 6 months after birth.
